# Multiple CD59 Polymorphisms in Chinese Patients with *Mycobacterium tuberculosis* Infection

**DOI:** 10.1155/2023/1216048

**Published:** 2023-04-02

**Authors:** Jie Tang, Zhenzhen Zhao, Juan Zhou, Lin Jiao, Wenjing Zhou, Binwu Ying, Yuwei Yang

**Affiliations:** ^1^Department of Laboratory Medicine, Mianyang Central Hospital, School of Medicine, University of Electronic Science and Technology of China, Mianyang 621000, China; ^2^Department of Laboratory Medicine, West China Hospital, Sichuan University, Chengdu 610041, China

## Abstract

**Methods:**

A case–control study was conducted to investigate the SNPs at CD59 rs1047581, rs7046, rs2231460, rs184251026, rs41275164, rs831633, rs704700, rs41275166, and rs10768024 by sequence-specific primer-polymerase chain reaction (SSP-PCR) in 900 tuberculosis patients and 1,534 controls.

**Results:**

The minor allele frequencies at rs2231460, rs184251026, rs41275164, and rs41275166 were extremely low both in the Cases (0.00%–0.61%) and in the Controls (0.07%–0.43%), comparatively at rs1047581, rs7046, rs831633, rs704700, and rs10768024 were notably higher both in the Cases (8.23%–48.39%) and in the Controls (8.57%–47.16%). Among the nine SNPs, only homozygous CC genotype at rs10768024 showed a significant protective effect against TB than homozygous TT genotype (OR(95% CI) = 0.59(0.38, 0.91), *χ*^2^ = 5.779, *P* = 0.016), and homozygous TT and heterozygous CT genotypes showed a significant risk of TB infection in the recessive model (OR(95% CI) = 1.68(1.10, 2.56), *χ*^2^ = 5.769, *P* = 0.016). Further analysis verified that rs10768024 CC genotype independently related to TB susceptibility (OR(95% CI) = 0.60(0.39, 0.91), Wald *χ*^2^ = 5.664, *P* = 0.017) in multivariate logistic regression analysis, and its genetic mutation was independent of the other SNPs (*r*^2^ = 0.00–0.20) in haplotype analysis.

**Conclusions:**

The first investigation of the CD59 gene and susceptibility to TB suggests a significant risk with homozygous TT and heterozygous CT genotypes at rs10768024 loci. The homozygous CC mutation at rs10768024 loci showed a significant protection against TB susceptibility.

## 1. Introduction

Tuberculosis (TB) is an arrantly infectious disease caused by *Mycobacterium tuberculosis* (MTB), and the No. 1 cause of death from infectious disease globally [1]. According to WHOs global progress report on tuberculosis elimination, an estimated 9.9 million people fell ill with TB worldwide in 2020, and in Asian countries, TB prevalence ranged from 119 per 100,000 population in China to 1,159 per 100,000 population in the Philippines [2]. Up to 2020, TB still has the highest mortality than other infectious diseases worldwide, killing 1.5 million people in 2018. In addition, the coronavirus (COVID-19) pandemic has caused enormous health, social, and economic impacts in 2020 and 2021 [3, 4], including essential TB services on prevention and cure, the number of diagnosing and notifying as TB cases through national disease surveillance systems, and TB incidence and mortality investigation.

Numerous genetic studies have shown that TB infections are the result of a combination of multiple genes and environmental factors in the virus and host [5–7]. TB susceptibility and progression depend on interactions between *Mycobacterium tuberculosis*, the host immune system, and genetic and environmental factors [8]. At first, the pathogenicity of MTB itself is crucial, and there are also differences among different strains. According to reports, thousands of single nucleotide polymorphisms (SNPs) in clinical MTB complex have been discovered by DNA sequencing [9, 10]. These genetic mutations reveal the differences and phylogenetic relationships between strains. Many of these mutations cause drug resistance while others can serve as phylogenetic markers for strain classification. But not only that, SNPs may have an impact on TB infection and disease outcome though the influence on the MTB phenotype in a variety of ways. Because of the diversity of MTBC populations and its SNPs, establishing a comprehensive database dedicated to SNP data of MTB strains is helpful to find the best fit between genomics and epidemiology of TB.

Individual differences in tuberculosis infection are related to host susceptibility genes [11, 12]. In recent years, host susceptibility genes and their SNPs have become research hotspots, which can provide a basis for the prevention and control, treatment and prognosis of TB, as well as for the new drug targets search [13]. TB is the result of the coaction of multiple genes while the distribution frequency of genic polymorphism varies in the race, population, and region [14–16], resulting in inconsistent or even contrary results obtained by many susceptibility genes and SNP in different populations or regions [8, 17, 18]. Therefore, it is necessary to further search for susceptibility genes that prevalently exist in the population and are exactly associated with TB.

Individual differences in TB infection are also related to the host immune system. Therefore, genetic factors related to immune system play an important role in tuberculosis. Different immune-related gene polymorphisms may attach different susceptibility to tuberculosis, such as these genes encoding VDR vitamin D receptor, mannose-binding lectin, human leukocyte antigen, Toll-like receptor, interleukin, interferon-*γ*, tumor necrosis factor-*α*, and immunity-related GTPase M [19–21]. But up to now, CD59 gene polymorphisms have never been discussed. The CD59 protein it encodes is an important complementary regulator that inhibits the formation of membrane attack complex and regulates signal transduction and T lymphocyte activation [22], and thus has become an indispensable part of the complement system. This plays an important immunomodulatory role in the process of TB infection and pathogenicity.

The complement system is a considerable first response of the host immune system, which plays an essential role in targeting MTB and MTB-infected cells and boosting the innate and acquired immune responses against MTB infection. Furthermore, to the best of our knowledge, there are no previous reports regarding CD59 SNPs' role in TB susceptibility in Chinese patients. Therefore, this study for the first time determined the association of CD59 polymorphisms with the susceptibility of TB.

## 2. Patients and Methods

### 2.1. Ethics

According to the principles of the Declaration of Helsinki, the protocol of this case–control study was approved by the Ethics Committee of Mianyang Central Hospital, affiliated with School of Medicine, University of Electronic Science and Technology of China (approval no. S2018085), and the Ethics Committee of West China Hospital, Sichuan University. The informed consents were obtained from all participants.

### 2.2. Patients

From January 2018 to December 2020, a total of 900 patients with newly diagnosed pulmonary and extrapulmonary TB were enrolled in West China Hospital, Sichuan University, and Mianyang Central Hospital affiliated with Medicine School of University of Electronic Science and Technology of China. Of all enrolled patients, there were 662 cases of pulmonary TB, 236 cases of extrapulmonary TB (including 87 cases of TB pleural effusion, 43 cases of TB Lymphadenitis, 11 cases of TB choroiditis, 13 cases of TB pericardial effusion, 8 cases of Pott's disease, 11 cases of TB ascites, 5 cases of TB cystitis, 25 cases of TB enteritis, 23 cases of TB mastitis, 8 cases of TB peritonitis, 4 cases of TB synovitis).

Inclusion criteria were as follows: (1) pulmonary TB was diagnosed by positive sputum smear and/or MTB culture [23]; (2) extrapulmonary TB was confirmed by histopathologic examination that the lesion sites were with emblematic character of tuberculous granulomas, which composed of multinucleated giant cells, epithelioid cells, and foam cells surrounded by a rim of lymphocytes [23]; (3) Pott's disease was diagnosed by magnetic resonance imaging examination, there was the presence of irregular endplate and anterior aspect of the vertebral bodies, accompanied by marrow edema and enhancement [24]; (4) TB choroiditis was characterized by deep, discrete, multiple, yellowish lesions ranging from 0.5 to 3.0 mm in diameter [25].

Exclusion criteria were as follows: (1) HIV-positive subjects; (2) previously treated TB cases before sample collection; (3) with any immune system diseases; (4) with severe cardiac, hepatic, and renal insufficiency; (5) with other serious infections; (6) pregnancy.

### 2.3. Controls

This study included 1,534 controls, whose age, sex, region, and ethnic background matched with the patients, and with no history of previous or current TB infection.

### 2.4. Sample Collection and DNA Extraction

Fasting venous blood samples from all participants were collected in a 3.0 ml ethylenediaminetetraacetic acid anticoagulation blood collection tubes (BD Vacutainers, Franklin Lakes, NJ, USA), moved upside down eight times for thorough mixing, and then DNA was extracted immediately or stored at −80°C for a maximum of 7 days. Genomic DNA extraction was carried out according to the operating instruction of a QIAamp DNA blood Mini kit (Qiagen, Hilden, Germany). DNA efficiency and quantity were detected by UV spectrophotometer after 0.8% agarose gel electrophoresis. The extracted DNA was stored at −80°C for a maximum of 6 months before further SNP analysis.

### 2.5. Candidate SNPs Selection and Genotyping

SNPs locus information of CD59 gene in a Chinese Han Beijing (CHB, Han Chinese in Beijing, China) were downloaded from IGSR and the 1000 Genomes Project (http://www.internationalgenome.org/vcf-ped-converter#online-version). Nine tagged SNPs (TagSNPs) were filtered by the pairwise tagger method with a threshold *r*^2^ > 0.8 systematically using the Haploview v4.2 software program, including rs1047581 C/T, rs7046 A/G, rs2231460 A/G, rs184251026 T/G, rs41275164 A/G, rs831633 A/G, rs704700 G/A, rs41275166 G/A, and rs10768024 C/T polymorphisms. But further according to the dbSNP database (https://www.ncbi.nlm.nih.gov/snp), only six CD59 polymorphisms with minor allele frequencies (MAF) >0.05 were retained. The retained polymorphisms were rs1047581 C/T, rs7046 A/G, rs831633 A/G, rs704700 G/A, and rs10768024 C/T polymorphisms, whose global MAFs were 0.29867/75214, 0.392832/93549, 0.39393/93032, 0.175056/44593, and 0.103897/26017, respectively. The eliminated polymorphisms were rs2231460 A/G, rs184251026 T/G, rs41275164 A/G, and rs41275166 G/A, whose global MAFs were 0.002769/733, 0.002685/45, 0.020881/5527, and 0.020865/886, respectively.

The SNP genotyping was performed by a custom-tailored 2 × 48-Plex SNPscan™ Kit (Genesky Biotechnologies Inc., Shanghai, China), which had been described in a previous study [26]. Its method was based on double ligation and multiplex fluorescence polymerase chain reaction. To further monitor the genotype quality, not only ddH_2_O was set as the negative control in each reaction, but also ∼10% of random samples were selected as repeat controls to genotype in duplicate with a concordance rate of 100%.

### 2.6. Statistical Analysis

Statistical analyses were performed using MedCalc 18.2 (MedCalc, Mariakerke, Belgium), PLINK 1.90 beta (GRAIL Inc., Menlo Park, USA), and SPSS 19.0 (SPSS Inc., Chicago, IL, USA) softwares. The continuous data were presented as M (P_25_, P_75_) (min, max), the difference analysis between the two groups was performed using the independent samples Mann–Whitney *U* test; the categorical data were presented as *n* or *n* (%), the difference analysis between two groups was performed using the *χ*^2^ test. Allele frequency and MAFs at CD59 genetic polymorphisms in either Cases or Controls group were presented in a form of composite cumulative area diagram and histogram, and the difference analysis between the two groups was performed by the *χ*^2^ test. The distribution comparison of allele, genotype, and genetic models (dominant and recessive model) was analyzed by bivariate or multivariate logistic regression analysis adjusted for age and gender using PLINK 1.90 beta software, and the association strength was evaluated with odds ratios (ORs) and corresponding 95% confidence intervals (CIs). *P* < 0.05 was considered to be statistically significant. Finally, multivariate logistic regression was used to analyze the interaction of multiple SNPs, and their association with tuberculosis susceptibility was evaluated via odds ratio (OR) with its 95% confidence interval (CI). The Haploview 4.2 freeware (Daly Lab, USA) was used to analyze the linkage disequilibrium and haplotype blocks of these SNPs, and the linkage disequilibrium degree was determined via the standardization coefficient (*D*′) and correlation coefficient (*r*^2^) of linkage disequilibrium.

## 3. Results

### 3.1. Demographic and Clinical Characteristics of the Study Participants

This study included 900 MTB patients (821 females and 41 males) with a median (P_25_, P_75_)/(min, max) age of 39(26, 53)/(14, 85) years and 1,534 controls (821 females and 58 males) with a median (P_25_, P_75_)/(min, max) age of 39(26, 53)/(14, 85) years. There were nonsignificant differences in age, sex, smoking status, and comorbidity between the Cases and the Controls (shown in Table 1). Pulmonary and extrapulmonary TB in the Cases represented 73.56% and 26.44%, respectively (shown in Table 2).

### 3.2. Minor Allele Frequency in Different Groups

**Figure 1:**
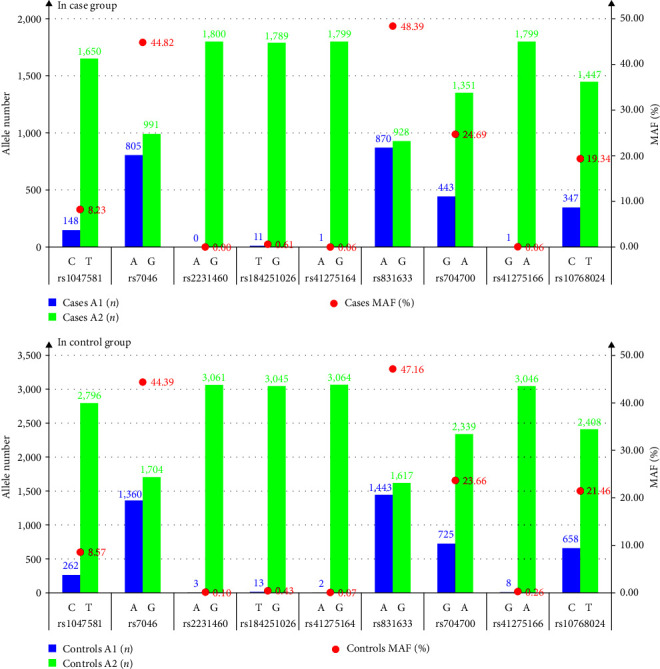
Composite graph of allele number histogram with MAF scatter plot. Note: In the label on the horizontal axis, the lower row is SNP, and the two alleles above are its minor allele (A1) and major allele (A2) in order; MAF, minor allele frequency. The histogram shows the number of alleles, and the red scatter shows the size of MAF. Among the nine SNPs of CD59 gene, the rates of allele mutation at rs1047581, rs7046, rs831633, rs704700, and rs10768024 loci were higher.

We investigated allele mutations in nine CD59 SNPs. Results showed that the SNP incidences in rs2231460, rs184251026, rs41275164, and rs41275166 were extremely low, with minor allele frequency (MAF) from 0.00% to 0.61% in the Cases (shown in Table 3 and Figure 1), and from 0.07% to 0.43% in the Controls (shown in Table 3 and Figure 1). Comparatively, the SNP incidences of rs1047581, rs7046, rs831633, rs704700, and rs10768024 were higher, with MAF from 8.23% to 48.39% in the Cases (shown in Table 3 and Figure 1), and from 8.57% to 47.16% in the Controls (shown in Table 3 and Figure 1). The incidences of all nine SNPs showed no significant difference between the Cases and the Controls (*χ*^2^ = 0.017–3.098, all *P* > 0.05).

### 3.3. Single Nucleotide Polymorphisms and Susceptibility to Tuberculosis

We compared the odds ratio of genotypes with one or two site mutations to the wild genotype and the mutation allele to the wild allele. Then we further analyzed the relationship between SNP and TB susceptibility by dominant and recessive models. Results showed (shown in Table 4) that among the nine SNPs of CD59 gene, only rs10768024 SNP was related to tuberculosis susceptibility, with OR(95% CI) = 0.59(0.38, 0.91) in CC/TT genotype comparison (*χ*^2^ = 5.779, *P* = 0.016), and OR(95% CI) = 1.68(1.10, 2.56) in recessive model (*χ*^*2*^ = 5.769, *P* = 0.016).

### 3.4. Multivariate Analysis of Susceptibility to Tuberculosis

The association of nine SNPs and tuberculosis susceptibility were analyzed by logistic regression method with different modes (shown in Table 5). The rs2231460, rs41275164, and rs41275166 were manually removed from the analysis due to the presence of multicollinearity. Of the remaining six SNPs, after adjusting for the demographic and clinical characteristics of participants, no SNPs were associated with tuberculosis susceptibility in the entry method logistic regression (Wald *χ*^2^ = 0.108–1.897, all *P* > 0.05) while only the homozygous CC alleles of rs10768024 was associated with tuberculosis susceptibility in the forward, backward, or stepwise logistic regression with OR(95% CI) = 0.60(0.39–0.91) (Wald *χ*^2^ = 5.664, *P* = 0.017). This indicated that the homozygous CC alleles of rs10768024 might be an independent factor for tuberculosis susceptibility among these SNPs.

### 3.5. Haplotype Analysis of Single Nucleotide Polymorphisms

**Figure 2:**
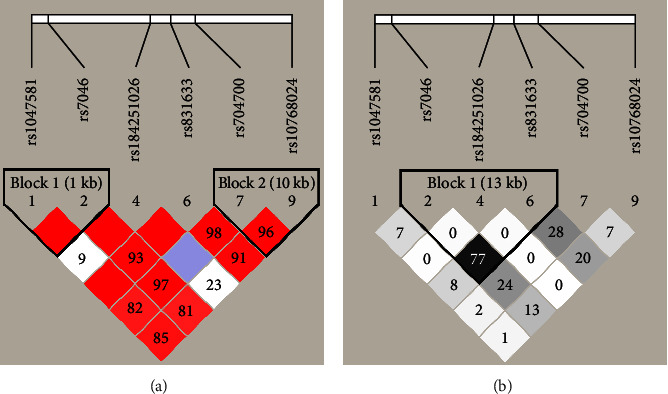
Linkage disequilibrium plot for the SNPs of CD59 gene. Note: (a) linkage disequilibrium decision with *D*′ value; (b) linkage disequilibrium decision with *r*^2^ value. Results showed that rs10768024 genetic mutation was almost no linkage disequilibrium with the other SNPs (*r*^2^ = 0.00–0.20).

We further analyzed the linkage disequilibrium and haplotype blocks of these SNPs. Of them, rs2231460, rs41275164, and rs41275166 were excluded from the analysis due to noncompliance with Hardy–Weinberg equilibrium or their MAF = 0.001. When using *D*′ value for deciding linkage disequilibrium, the six SNPs were tagged into two haplotype blocks by the Gabriel method (shown in Figure 2(a)), rs1047581 was in complete linkage disequilibrium with rs7046 (*D*′ = 1.00) and rs10768024 was in strong linkage disequilibrium with rs704700 (*D*′ = 0.96). But when using *r*^2^ value for further haplotype analysis (shown in Figure 2(b)), only rs7046, rs184251026, and rs831633 could be barely tagged into a haplotype block (*r*^2^ = 0.77) while rs10768024 was not in linkage disequilibrium with the other RNPs (*r*^2^ = 0.00–0.20). This result indicated that its genetic mutation was completely or largely independent of the other SNPs.

## 4. Discussion

TB disease progression is heterogeneous and circuitous, and its mechanism is unclear [27, 28]. In recent years, due to environmental pollution, population mobility, AIDS spread, and especially COVID-19 ravage, the TB incidence in developing countries is much higher than the global average [29, 30]. After the host is infected with MTB, whether MTB can reside in the body is closely related to the number and virulence of infected bacteria, as well as cellular immune function of the host [31, 32]. Genetic factors also play an important role in the occurrence and development of TB. With the deepening of genetic susceptibility gene research on TB, it has been found that numerous SNPs are associated with tuberculosis susceptibility [21, 33, 34]. But so far, no study has reported whether CD59 SNPs are associated with TB susceptibility. This first study on CD59 genetic SNPs and tuberculosis susceptibility found that the TT and CT genotypes containing the ancestral allele at rs10768024 loci had a significant risk of TB susceptibility. The homozygous CC allele mutation at rs10768024 loci showed a significant protective effect against MTB infection than the homozygous TT and heterozygous CT genotypes. Further multivariate interaction analysis and haplotype analysis showed that rs10768024 genetic mutation was completely or largely independent of the other SNPs, and homozygous CC mutation might be an independent factor for tuberculosis susceptibility.

CD59 is a glycoprotein anchored to the surface of cell membranes via glycosyl phosphatidyl inositol (GPI). It was discovered and reported in 1988 by Sugita et al. [35], also known as homologous restriction factor (HRF20), C8 binding protein (CBbp), membrane inhibitor of reactive lysis (MIRL), and Protectin [36]. The human CD59 gene is located on the short arm of chromosome 11 (11p13), with about 40 kb of DNA size, containing six exons and six introns (NG_008057) [37]. At present, a total of eight related mRNA sequences have been stored in the NCBI database, encoding a single chain containing 128 amino acids. Its 25 amino acids in front of the N-terminal are signal peptides, which can be identified, cleaved, and replaced via a preassembled GPI; and its 26 amino acids at the C-terminal are hydrophobic terminal, which are removed when attaching to the GPI anchor. The mature CD59 protein contained 77 amino acids.

CD59 is a multifunctional protein, and two of its most important functions are as follows: one is that in the terminal stage of complement cascade, CD59 can prevent the formation of complement membrane attack complex via interfering with the interaction of C8 and/or C9, the C9 polymerization, or the C9 insertion into cell membrane, thus preventing the construction of the first defense line of innate immunity (that is complement defense system), so as to negatively regulate the activation of the complement system [38]; another is that as one of the natural ligands for CD2, CD59 is involved in T-cell activation and adhesion via activating p56^Lck^ and P70^ZAP^ or activating protease C [39]. Therefore, in terms of function, CD59 molecule has dual properties of protecting or resisting MTB infection. After MTB infection, it is often parasitized to host mononuclear macrophages to replicate and persist [40]. Animal experiments or clinical studies have confirmed that CD59 can maintain the host immune homeostasis and contribute to complement resistance after inflammatory injury [41], tumorigenesis [42], and viral infection [43]. This is conducive to TB susceptibility. We also found that in the recessive model, the reservation of ancestral allele T at one or two rs10768024 loci increased TB susceptibility 1.68 times, and only homozygous CC genotype showed an opposability against tuberculosis susceptibility. Thus, the capacity to combat TB susceptibility may come from the homozygous CC genotype alone, that is T⟶C mutation occurred at both rs10768024 loci.

According to the International Genome Sample Resource (IGSR) website, SNP rs10768024 is an intron nucleotide sequence and its T⟶C variant is intron variant. We infer that ancestral allele T reservation at rs10768024 loci can ensure the normal regulation of CD59 transcription, which is important for translating into normal CD59 molecules and maintaining their function. The TB susceptibility from homozygous TT and heterozygous CT genotypes may be related to the prevention of the formation of membrane attack complex by CD59. When T⟶C mutation occurs in both alleles of rs10768024, CD59 variant is unable to effectively prevent the formation of membrane attack complex. Therefore, it shows the ability to fight against MTB infection. However, further cell experiments, animal models, or genetic analysis are needed to clarify these.

Another important function of CD59 is involved in the early events of T-cell activation as the second ligand of CD2, which seems to be detrimental to the persistence of MTB infection. The binding of CD59 and CD2 can enhance the adhesion between T cells and other cells, promote the activation of T cells, and enhance the action of other immune cells. In clinical practice, we also pay attention to the elevated level of CD59 in tuberculosis patients, which is expressed not only in monocyte–macrophages but also in T cells. These events of CD59 promoting T-cell activation and increased expression on T cells suggest that CD59 may promote fighting against MTB infection. However, few studies have suggested a direct inhibitory role of CD59 on T-cell activation by a complement-independent manner, as demonstrated by the increased CD4+ T-cell responses in CD59-knockout mice, but was not influenced in CD8+ T-cell activation [44, 45]. This inhibiting effect on CD4+ T-cell activation leads to the weakening of cellular immune response and the relative enhancing of humoral immunity mediated by CD8+ T-cell. We guess that this may be one of the mechanisms of MTB immune escape. Unfortunately, studies on the relationship between MTB and CD59 gene, mRNA, or protein are still lacking. Therefore, whether the effect of CD59 on T cells is beneficial or detrimental to the persistence of MTB infection still needs more research to reveal.

## 5. Conclusion

In conclusion, this study for the first time revealed the relationship between CD59 genomic polymorphisms and TB susceptibility in Chinese. Our study suggests that rs10768024 TT and CT genotypes have one or two T ancestral alleles, with obvious TB susceptibility. The CC genotype mutation is a possible adaptive change of the host against MTB infection.

## Figures and Tables

**Table 1:** Basic characteristics of the study participants.

Characteristics variable	Cases (*n* = 900)	Controls (*n* = 1,534)	*z*/*χ*^2^	*P*-value
Age (years)^*∗*^			1.326	0.185
Median	39	36		
(P_25_, P_75_)	(26, 53)	(29, 45)		
(Min, max)	(14, 85)	(17, 80)		
Sex (*n* (%))			2.759	0.097
Male	542 (60.2)	871 (56.8)		
Female	358 (39.8)	663 (43.2)		
Smoking status (*n* (%))			3.431	0.180
Nonsmoker	592 (65.8)	1055 (68.8)		
Ex-smoker	237 (26.3)	377 (24.6)		
Current smoker	71 (7.9)	101 (6.6)		
Comorbidity (*n* (%))			7.249	0.298
None	579 (64.3)	1032 (67.3)		
HTN	138 (15.3)	185 (12.1)		
Malignant mesothelioma	17 (1.9)	25 (1.6)		
HCV	25 (2.8)	57 (3.7)		
DM	95 (10.6)	160 (10.4)		
Asthma	5 (0.6)	10 (0.7)		
Ischemic heart disease	41 (4.6)	65 (4.2)		

Note:  ^*∗*^Tested by independent samples Mann–Whitney test, and the others tested by *χ*^2^ test. DM, diabetes mellitus; HTN, hypertension; HCV, viral hepatitis C.

**Table 2:** Distribution of affected sites with MTB infection in the study participants.

Diagnosis	Frequency	Percentage (%)
Pulmonary TB	662/900	73.56
Extrapulmonary TB	238/900	26.44
TB pleural effusion	87/238	36.55
TB lymphadenitis	43/238	18.07
TB choroiditis	11/238	4.62
TB pericardial effusion	13/238	5.46
Pott's disease	8/238	3.36
TB ascites	11/238	4.62
TB cystitis	5/238	2.10
TB enteritis	25/238	10.50
TB mastitis	23/238	9.66
TB peritonitis	8/238	3.36
TB synovitis	4/238	1.68

**Table 3:** The minor allele frequency of nine SNP in CD59 gene.

SNP	A1	A2	Cases			Controls			*χ* ^2^ test		
			A1 (*n*)	A2 (*n*)	MAF (%)	A1 (*n*)	A2 (*n*)	MAF (%)	*χ* ^2^	P1	P2
rs1047581	C	T	148	1,650	8.23	262	2,796	8.57	0.166	0.684	0.738
rs7046	A	G	805	991	44.82	1,360	1704	44.39	0.087	0.768	0.808
rs2231460	A	G	0	1,800	0.00	3	3,061	0.10	1.763	0.184	0.274
rs184251026	T	G	11	1,789	0.61	13	3,045	0.43	0.797	0.372	0.462
rs41275164	A	G	1	1,799	0.06	2	3,064	0.07	0.017	0.896	0.914
rs831633	A	G	870	928	48.39	1,443	1617	47.16	0.687	0.407	0.495
rs704700	G	A	443	1,351	24.69	725	2,339	23.66	0.659	0.417	0.504
rs41275166	G	A	1	1,799	0.06	8	3,046	0.26	2.607	0.106	0.204
rs10768024	C	T	347	1,447	19.34	658	2,408	21.46	3.098	0.078	0.148

Note: A1, minor allele; A2, major allele; MAF, minor allele frequency. P1, *P*-value of Pearson *χ*^2^ test; P2, *P*-value of correction for continuity *χ*^2^ test.

**Table 4:** The relevance between single nucleotide polymorphisms and susceptibility to tuberculosis.

SNP	Test	Comparison	Cases	Controls	OR(95% CI)	*χ* ^*2*^	*P*-value
rs1047581	Genotypes	CT/TT	138/756	240/1,278	0.97(0.77, 1.22)	0.004	0.807
(*n* = 2428)		CC/TT	5/756	11/1,278	0.77(0.27, 2.22)	0.056	0.627
	Alleles	C/T	148/1,650	262/2,796	0.96(0.78, 1.18)	0.166	0.684
	DOM	(CC + CT)/TT	143/756	251/1,278	0.96(0.77, 1.21)	0.108	0.742
	REC	(CT + TT)/CC	894/5	1518/11	1.29(0.45, 3.74)	0.231	0.631
rs7046	Genotypes	AG/GG	433/279	754/475	0.98(0.81, 1.18)	0.055	0.815
(*n* = 2430)		AA/GG	186/279	303/475	1.05(0.83, 1.32)	0.135	0.713
	Alleles	A/G	805/991	1,360/1,704	1.02(0.91, 1.14)	0.087	0.768
	DOM	(AG + AA)/GG	619/279	1,057/475	1.00(0.83, 1.19)	0.001	0.974
	REC	(AG + GG)/AA	712/186	1,229/303	0.94(0.77, 1.16)	0.308	0.579
rs2231460	Genotypes	AG/GG	0/900	3/1,529	0.24(0.01, 4.70)	0.876	0.349
(*n* = 2432)		AA/GG	0/900	0/1,529	1.70(0.03, 85.68)	0.070	0.791
	Alleles	A/G	0/1,800	3/3,061	0.24(0.01, 4.71)	1.763	0.184
	DOM	(AG + AA)/GG	0/900	3/1,529	0.24(0.01, 4.70)	0.876	0.349
	REC	(AG + GG)/AA	900/0	1,532/0	1.70(0.03, 85.85)	0.071	0.791
rs184251026	Genotypes	TG/GG	11/889	13/1,516	1.44(0.64, 3.23)	0.794	0.373
(*n* = 2329)		TT/GG	0/889	0/1,516	1.70(0.03, 86.00)	0.071	0.789
	Alleles	T/G	11/1,789	13/3,045	1.44(0.64, 3.22)	0.797	0.372
	DOM	(TT + TG)/GG	11/889	13/1,516	1.44(0.64, 3.24)	0.890	0.365
	REC	(GG + TG)/TT	900/0	1,529/0	1.70(0.03, 85.68)	0.070	0.791
rs41275164	Genotypes	AG/GG	1/899	2/1,531	0.86(0.08, 9.51)	0.015	0.903
(*n* = 2433)		AA/GG	0/899	0/1,531	1.72(0.03, 86.85)	0.074	0.786
	Alleles	A/G	1/1,799	2/3,064	0.85(0.08, 9.40)	0.017	0.896
	DOM	(AG + AA)/GG	1/899	2/1,531	0.85(0.08, 9.40)	0.017	0.896
	REC	(AG + GG)/AA	900/0	1,533/0	1.70(0.03, 85.90)	0.071	0.791
rs831633	Genotypes	AG/GG	434/247	743/437	1.03(0.85, 1.26)	0.108	0.742
(*n* = 2429)		AA/GG	218/247	350/437	1.10(0.88, 1.39)	0.684	0.408
	Alleles	A/G	870/928	1,443/1617	1.05(0.93, 1.18)	0.687	0.407
	DOM	(AG + AA)/GG	652/247	1,093/437	1.06(0.88, 1.27)	0.331	0.565
	REC	(AG + GG)/AA	681/218	1,180/350	0.92(0.76, 1.12)	0.596	0.440
rs704700	Genotypes	GA/AA	351/500	545/897	1.16(0.97, 1.37)	2.676	0.102
(*n* = 2429)		GG/AA	46/500	90/897	0.92(0.63, 1.33)	0.209	0.648
	Alleles	G/A	443/1,351	725/2,339	1.06(0.92, 1.21)	0.659	0.417
	DOM	(AG + GG)/AA	397/500	635/897	1.12(0.95, 1.33)	1.828	0.176
	REC	(AG + AA)/GG	851/46	1,442/90	1.15(0.80, 1.66)	0.596	0.440
rs41275166	Genotypes	GA/AA	1/899	2/1,522	0.85(0.08, 9.35)	0.018	0.892
(*n* = 2427)		GG/AA	0/899	3/1,522	0.24(0.01, 4.69)	0.882	0.348
	Alleles	G/A	1/1,799	8/3,046	0.21(0.03, 1.69)	2.140	0.143
	DOM	(AG + GG)/AA	1/899	5/1,522	0.34(0.04, 2.90)	0.976	0.323
	REC	(AG + AA)/GG	900/0	1,524/3	4.13(0.21, 80.14)	0.882	0.348
rs10768024	Genotypes	CT/TT	287/580	490/959	0.97(0.81, 1.16)	0.124	0.725
(*n* = 2430)		CC/TT	30/580	84/959	0.59(0.38, 0.91)	5.779	0.016
	Alleles	C/T	347/1,447	658/2,408	0.88(0.76, 1.02)	3.098	0.078
	DOM	(CT + CC)/TT	317/580	574/959	0.91(0.77, 1.08)	1.078	0.299
	REC	(CT + TT)/CC	867/30	1,449/84	1.68(1.10, 2.56)	5.769	0.016

Note: SNP, single nucleotide polymorphisms; DOM, dominant model; REC, recessive model. Individuals with missing data were excluded.

**Table 5:** Multivariate logistic regression analysis with different modes for the nine SNPs.

Alleles	Entry logistic regression				Forward, backward, or stepwise logistic regression			
	OR	95% CI	Wald *χ*^2^	*P*-value	OR	95% CI	Wald *χ*2	*P*-value
rs1047581 CT	0.92	0.60–1.41	0.143	0.705	–	–	–	–
rs1047581 CC	0.65	0.18–2.34	0.432	0.511	–	–	–	–
rs7046 AG	0.81	0.56–1.15	1.411	0.235	–	–	–	–
rs7046 AA	0.80	0.48–1.33	0.747	0.387	–	–	–	–
rs184251026 TG	1.54	0.68–3.48	1.073	0.300	–	–	–	–
rs831633 AG	1.06	0.65–1.75	0.060	0.806	–	–	–	–
rs831633 AA	1.16	0.50–2.68	0.118	0.732	–	–	–	–
rs704700 GA	1.08	0.72–1.63	0.133	0.716	–	–	–	–
rs704700 GG	0.77	0.34–1.77	0.378	0.539	–	–	–	–
rs10768024 CT	0.94	0.63–1.40	0.108	0.742	–	–	–	–
rs10768024 CC	0.56	0.24–1.28	1.897	0.168	0.60	0.39–0.91	5.664	0.017

Note: Adjusted for age, sex, smoking status, and comorbidity. “–” indicates removal from logistic regression analysis due to *P* > 0.1.

## Data Availability

The datasets used and analyzed during the current study are available from the corresponding author on reasonable request.

## Additional Points

*Limitations of the Study*. This study is the first report on the relationship between CD59 genomic polymorphisms and TB susceptibility in the Chinese Han population. More studies are needed to confirm whether the conclusion is correct and whether it is applicable to other ethnic groups. This study only revealed the relationship between rs10768024 SNP and TB susceptibility, and its mechanism remains unclear, which needs further cell experiments, animal models, and clinical studies to confirm. This will expand a new idea and approach for the prevention and treatment of MTB infection.
